# 3-(2-Hydr­oxy-5-methyl­anilino)isobenzo­furan-1(3*H*)-one^1^

**DOI:** 10.1107/S1600536808008441

**Published:** 2008-04-02

**Authors:** Mustafa Odabaşoğlu, Orhan Büyükgüngör

**Affiliations:** a Department of Chemistry, Faculty of Arts & Sciences, Ondokuz Mayıs University, TR-55139 Kurupelit Samsun, Turkey; b Department of Physics, Faculty of Arts & Sciences, Ondokuz Mayıs University, TR-55139 Kurupelit Samsun, Turkey

## Abstract

In the mol­ecule of the title compound, C_15_H_13_NO_3_, the phthalide ring system is virtually planar, with a dihedral angle of 1.98 (3)° between the fused five- and six-membered rings. The substituted aromatic ring is oriented at a dihedral angle of 57.50 (3)° with respect to the phthalide ring system. In the crystal structure, inter­molecular O—H⋯O and N—H⋯O hydrogen bonds link the mol­ecules, forming a three-dimensional network.

## Related literature

For a related structure, see: Odabaşoğlu & Büyükgüngör (2006 ▶). For ring-motif details, see: Bernstein *et al.* (1995 ▶); Etter (1990 ▶).
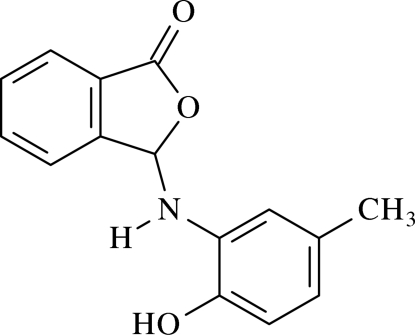


## Experimental

### 

#### Crystal data

C_15_H_13_NO_3_
*M* *_r_* = 255.26
Orthorhombic, 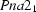

*a* = 8.7198 (5) Å
*b* = 15.5950 (14) Å
*c* = 9.3992 (6) Å
*V* = 1278.15 (16) Å^3^
*Z* = 4
Mo *K*α radiation
μ = 0.09 mm^−1^
*T* = 296 K
0.64 × 0.42 × 0.28 mm

#### Data collection

Stoe IPDSII diffractometer
Absorption correction: integration (*X-RED32*; Stoe & Cie, 2002 ▶) *T*_min_ = 0.957, *T*_max_ = 0.982
4658 measured reflections
1418 independent reflections
1196 reflections with *I* > 2σ(*I*)
*R*_int_ = 0.027

#### Refinement

*R*[*F* ^2^ > 2σ(*F* ^2^)] = 0.028
*wR*(*F* ^2^) = 0.066
*S* = 0.99
1418 reflections
175 parameters
1 restraint
H-atom parameters constrained
Δρ_max_ = 0.08 e Å^−3^
Δρ_min_ = −0.09 e Å^−3^

### 

Data collection: *X-AREA* (Stoe & Cie, 2002 ▶); cell refinement: *X-AREA*; data reduction: *X-RED32* (Stoe & Cie, 2002 ▶); program(s) used to solve structure: *SHELXS97* (Sheldrick, 2008 ▶); program(s) used to refine structure: *SHELXL97* (Sheldrick, 2008 ▶); molecular graphics: *ORTEP-3 for Windows* (Farrugia, 1997 ▶); software used to prepare material for publication: *WinGX* (Farrugia, 1999 ▶).

## Supplementary Material

Crystal structure: contains datablocks I, global. DOI: 10.1107/S1600536808008441/hk2444sup1.cif

Structure factors: contains datablocks I. DOI: 10.1107/S1600536808008441/hk2444Isup2.hkl

Additional supplementary materials:  crystallographic information; 3D view; checkCIF report

## Figures and Tables

**Table 1:** Hydrogen-bond geometry (Å, °)

*D*—H⋯*A*	*D*—H	H⋯*A*	*D*⋯*A*	*D*—H⋯*A*
O3—H3*A*⋯O1^i^	0.82	1.95	2.767 (2)	173
N1—H1⋯O2^ii^	0.86	2.78	3.5593 (19)	152

Symmetry codes: (i) 
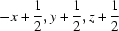
; (ii) 
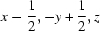
.

**Fig. 1.**
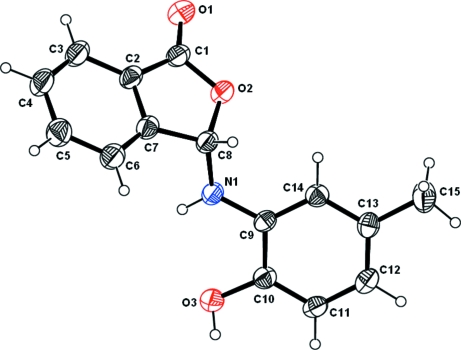
The molecular structure of the title molecule, with the atom-numbering scheme. Displacement ellipsoids are drawn at the 30% probability level.

**Fig. 2.**
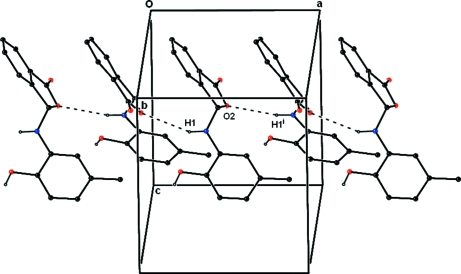
A partial packing diagram of (I), showing the formation of C(4) chain along the [100] direction. Hydrogen bonds are shown as dashed lines [symmetry code: (i) 1 - x, -y, z + 1/2]. H atoms not involved in hydrogen bondings have been omitted for clarity.

**Fig. 3.**
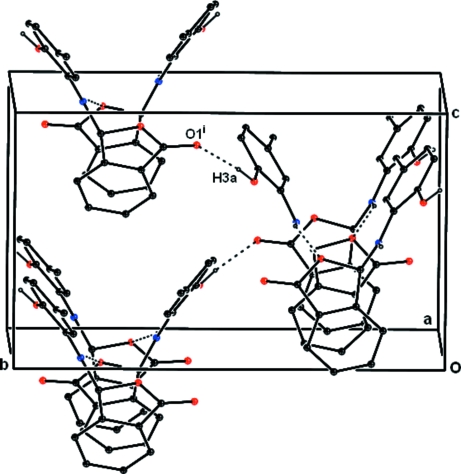
A partial packing diagram of (I). Hydrogen bonds are shown as dashed lines [symmetry code: (i) 1 - x, -y, z + 1/2]. H atoms not involved in hydrogen bondings have been omitted for clarity.

## supplementary crystallographic information

### Comment

The present work is part of a structural study of compounds of 3-substituted phthalides, and we report here the crystal structure of the title compound, (I).

The molecule of (I), (Fig. 1), is built up from a phthalimide unit connected to 2-hydroxy-5-methylphnyl group through an amino group. Rings A (C2-C7), B (C1/C2/C7/C8/O2) and C (C9-C14) are, of course, planar. The dihedral angles between them are A/B = 1.98 (3)°, A/C = 58.27 (3)° and B/C = 56.39 (3)°. So, rings A and B are also nearly coplanar. Ring C is oriented with respect to the coplanar ring system at a dihedral angle of 57.50 (3)°.

In the crystal structure, intermolecular O-H···O and N-H···O hydrogen bonds (Table 1) link the molecules by C(4) chains (Fig. 2) (Bernstein *et al.*, 1995; Etter, 1990), to form a three-dimensional network (Fig. 3), in which they may be effective in the stabilization of the structure.

### Experimental

The title compound was prepared according to the method described by Odabaşoğlu & Büyükgüngör (2006), using phthalaldehydic acid and 2-aminophenol as starting materials (yield; 80%). Crystals of (I) suitable for X-ray analysis were obtained by slow evaporation of an ethanol-DMF (1:1) solution at room temperature.

### Refinement

H atoms were positioned geometrically, with O-H = 0.82 Å (for OH), N-H = 0.86 Å (for NH) and C-H = 0.93 and 0.98 Å for aromatic and methine H, respectively, and constrained to ride on their parent atoms with U_iso_(H) = xU_eq_(C,O,N), where x = 1.5 for OH and NH H and x = 1.2 for all other H atoms.

### Crystal data

**Table:**

C_15_H_13_NO_3_	*F*_000_ = 536
*M**_r_* = 255.26	*D*_x_ = 1.327 Mg m^−^^3^
Orthorhombic, *P**n**a*2_1_	Mo *K*α radiation λ = 0.71073 Å
Hall symbol: P 2c -2n	Cell parameters from 4658 reflections
*a* = 8.7198 (5) Å	θ = 2.2–27.2º
*b* = 15.5950 (14) Å	µ = 0.09 mm^−^^1^
*c* = 9.3992 (6) Å	*T* = 296 K
*V* = 1278.15 (16) Å^3^	Prism, colorless
*Z* = 4	0.64 × 0.42 × 0.28 mm

### Data collection

**Table:**

Stoe IPDSII diffractometer	1418 independent reflections
Monochromator: plane graphite	1196 reflections with *I* > 2σ(*I*)
Detector resolution: 6.67 pixels mm^-1^	*R*_int_ = 0.027
*T* = 296 K	θ_max_ = 26.7º
ω rotation method scans	θ_min_ = 2.5º
Absorption correction: integration(X-RED32; Stoe & Cie, 2002)	*h* = −11→10
*T*_min_ = 0.957, *T*_max_ = 0.982	*k* = −12→19
4658 measured reflections	*l* = −10→11

### Refinement

**Table:**

Refinement on *F*^2^	Secondary atom site location: difference Fourier map
Least-squares matrix: full	Hydrogen site location: inferred from neighbouring sites
*R*[*F*^2^ > 2σ(*F*^2^)] = 0.028	H-atom parameters constrained
*wR*(*F*^2^) = 0.066	*w* = 1/[σ^2^(*F*_o_^2^) + (0.0415*P*)^2^] where *P* = (*F*_o_^2^ + 2*F*_c_^2^)/3
*S* = 0.99	(Δ/σ)_max_ < 0.001
1418 reflections	Δρ_max_ = 0.08 e Å^−^^3^
175 parameters	Δρ_min_ = −0.08 e Å^−^^3^
1 restraint	Extinction correction: SHELXL97 (Sheldrick, 2008), Fc^*^=kFc[1+0.001xFc^2^λ^3^/sin(2θ)]^-1/4^
Primary atom site location: structure-invariant direct methods	Extinction coefficient: 0.014 (2)

### Special details

**Table:**

Geometry. All e.s.d.'s (except the e.s.d. in the dihedral angle between two l.s. planes) are estimated using the full covariance matrix. The cell e.s.d.'s are taken into account individually in the estimation of e.s.d.'s in distances, angles and torsion angles; correlations between e.s.d.'s in cell parameters are only used when they are defined by crystal symmetry. An approximate (isotropic) treatment of cell e.s.d.'s is used for estimating e.s.d.'s involving l.s. planes.
Refinement. Refinement of F^2^ against ALL reflections. The weighted R-factor wR and goodness of fit S are based on F^2^, conventional R-factors R are based on F, with F set to zero for negative F^2^. The threshold expression of F^2^ > 2sigma(F^2^) is used only for calculating R-factors(gt) etc. and is not relevant to the choice of reflections for refinement. R-factors based on F^2^ are statistically about twice as large as those based on F, and R- factors based on ALL data will be even larger.

### Fractional atomic coordinates and isotropic or equivalent isotropic displacement parameters (Å^2^)

**Table:**

	*x*	*y*	*z*	*U*_iso_*/*U*_eq_	
O1	0.40903 (18)	0.07832 (10)	0.36000 (19)	0.0720 (4)	
O2	0.45824 (13)	0.20974 (10)	0.43987 (15)	0.0570 (4)	
O3	0.20122 (14)	0.44221 (12)	0.70125 (18)	0.0703 (5)	
H3A	0.1679	0.4796	0.7542	0.105*	
N1	0.34673 (16)	0.34164 (11)	0.51886 (18)	0.0520 (4)	
H1	0.2484	0.3454	0.5210	0.062*	
C1	0.3901 (2)	0.15492 (14)	0.3495 (2)	0.0545 (5)	
C2	0.3001 (2)	0.20426 (14)	0.2462 (2)	0.0525 (5)	
C3	0.2126 (2)	0.17580 (17)	0.1316 (3)	0.0669 (6)	
H3	0.2014	0.1177	0.1121	0.080*	
C4	0.1436 (3)	0.23701 (19)	0.0486 (3)	0.0749 (7)	
H4	0.0837	0.2199	−0.0283	0.090*	
C5	0.1609 (3)	0.32357 (18)	0.0765 (3)	0.0726 (6)	
H5	0.1146	0.3636	0.0169	0.087*	
C6	0.2468 (2)	0.35171 (17)	0.1926 (2)	0.0631 (6)	
H6	0.2576	0.4098	0.2129	0.076*	
C7	0.31506 (19)	0.28990 (15)	0.2760 (2)	0.0517 (5)	
C8	0.41502 (19)	0.30062 (13)	0.4044 (2)	0.0495 (4)	
H8	0.5076	0.3322	0.3771	0.059*	
C9	0.43056 (19)	0.37720 (11)	0.6310 (2)	0.0433 (4)	
C10	0.35434 (19)	0.43126 (13)	0.7257 (2)	0.0491 (4)	
C11	0.4335 (2)	0.46949 (13)	0.8349 (2)	0.0533 (5)	
H11	0.3819	0.5041	0.8997	0.064*	
C12	0.5902 (2)	0.45668 (13)	0.8491 (2)	0.0548 (5)	
H12	0.6432	0.4842	0.9217	0.066*	
C13	0.6683 (2)	0.40359 (14)	0.7568 (2)	0.0511 (4)	
C14	0.5859 (2)	0.36305 (13)	0.6500 (2)	0.0493 (4)	
H14	0.6364	0.3253	0.5895	0.059*	
C15	0.8385 (2)	0.3893 (2)	0.7713 (3)	0.0793 (7)	
H15A	0.8895	0.4095	0.6872	0.119*	
H15B	0.8584	0.3292	0.7833	0.119*	
H15C	0.8760	0.4201	0.8526	0.119*	

### Atomic displacement parameters (Å^2^)

**Table:**

	*U*^11^	*U*^22^	*U*^33^	*U*^12^	*U*^13^	*U*^23^
O1	0.0755 (9)	0.0573 (10)	0.0832 (11)	−0.0036 (7)	−0.0069 (9)	−0.0174 (9)
O2	0.0564 (6)	0.0588 (9)	0.0558 (7)	−0.0021 (6)	−0.0041 (7)	−0.0153 (7)
O3	0.0509 (7)	0.0807 (12)	0.0793 (10)	0.0085 (7)	−0.0030 (8)	−0.0320 (8)
N1	0.0421 (7)	0.0615 (10)	0.0523 (9)	−0.0042 (7)	0.0017 (7)	−0.0164 (8)
C1	0.0486 (9)	0.0570 (12)	0.0580 (11)	−0.0076 (9)	0.0066 (9)	−0.0176 (11)
C2	0.0469 (9)	0.0619 (13)	0.0488 (10)	−0.0091 (8)	0.0069 (8)	−0.0155 (10)
C3	0.0619 (11)	0.0785 (17)	0.0603 (12)	−0.0107 (11)	−0.0022 (10)	−0.0241 (13)
C4	0.0698 (13)	0.095 (2)	0.0599 (13)	−0.0043 (12)	−0.0101 (11)	−0.0271 (14)
C5	0.0718 (13)	0.0891 (19)	0.0571 (12)	0.0046 (12)	−0.0071 (10)	−0.0059 (12)
C6	0.0643 (12)	0.0654 (15)	0.0597 (12)	−0.0035 (10)	−0.0028 (10)	−0.0063 (11)
C7	0.0454 (9)	0.0633 (13)	0.0465 (10)	−0.0068 (8)	0.0053 (8)	−0.0110 (10)
C8	0.0457 (8)	0.0528 (11)	0.0501 (11)	−0.0061 (8)	0.0012 (8)	−0.0103 (9)
C9	0.0503 (9)	0.0392 (10)	0.0405 (8)	−0.0068 (7)	0.0015 (8)	−0.0007 (8)
C10	0.0501 (9)	0.0467 (11)	0.0506 (10)	−0.0026 (7)	0.0022 (8)	−0.0032 (9)
C11	0.0655 (11)	0.0466 (11)	0.0480 (10)	0.0007 (8)	−0.0011 (9)	−0.0087 (9)
C12	0.0683 (11)	0.0502 (11)	0.0460 (10)	−0.0103 (9)	−0.0089 (9)	−0.0013 (10)
C13	0.0526 (9)	0.0551 (12)	0.0456 (10)	−0.0060 (9)	−0.0040 (9)	0.0050 (9)
C14	0.0502 (9)	0.0523 (12)	0.0453 (10)	−0.0004 (8)	0.0044 (8)	−0.0021 (9)
C15	0.0531 (11)	0.115 (2)	0.0695 (14)	−0.0051 (12)	−0.0079 (11)	−0.0088 (15)

### Geometric parameters (Å, °)

**Table:**

O3—H3A	0.8200	C8—O2	1.504 (3)
N1—H1	0.8600	C8—H8	0.9800
C1—O1	1.210 (3)	C9—C14	1.384 (2)
C1—O2	1.344 (2)	C9—C10	1.394 (3)
C1—C2	1.466 (3)	C9—N1	1.397 (2)
C2—C7	1.371 (3)	C10—O3	1.366 (2)
C2—C3	1.393 (3)	C10—C11	1.373 (3)
C3—C4	1.372 (4)	C11—C12	1.387 (3)
C3—H3	0.9300	C11—H11	0.9300
C4—C5	1.383 (4)	C12—C13	1.379 (3)
C4—H4	0.9300	C12—H12	0.9300
C5—C6	1.395 (3)	C13—C14	1.387 (3)
C5—H5	0.9300	C13—C15	1.506 (3)
C6—C7	1.378 (3)	C14—H14	0.9300
C6—H6	0.9300	C15—H15A	0.9600
C7—C8	1.498 (3)	C15—H15B	0.9600
C8—N1	1.386 (2)	C15—H15C	0.9600
			
O1—C1—O2	121.1 (2)	C14—C9—N1	123.10 (16)
O1—C1—C2	130.19 (19)	C10—C9—N1	118.16 (15)
O2—C1—C2	108.75 (18)	O3—C10—C11	124.26 (17)
C7—C2—C3	121.4 (2)	O3—C10—C9	115.74 (16)
C7—C2—C1	108.97 (16)	C11—C10—C9	120.00 (16)
C3—C2—C1	129.7 (2)	C10—C11—C12	120.27 (18)
C4—C3—C2	117.3 (2)	C10—C11—H11	119.9
C4—C3—H3	121.4	C12—C11—H11	119.9
C2—C3—H3	121.4	C13—C12—C11	120.84 (17)
C3—C4—C5	121.5 (2)	C13—C12—H12	119.6
C3—C4—H4	119.2	C11—C12—H12	119.6
C5—C4—H4	119.2	C12—C13—C14	118.20 (16)
C4—C5—C6	120.9 (2)	C12—C13—C15	121.22 (18)
C4—C5—H5	119.5	C14—C13—C15	120.58 (19)
C6—C5—H5	119.5	C9—C14—C13	121.89 (17)
C7—C6—C5	117.2 (2)	C9—C14—H14	119.1
C7—C6—H6	121.4	C13—C14—H14	119.1
C5—C6—H6	121.4	C13—C15—H15A	109.5
C2—C7—C6	121.62 (19)	C13—C15—H15B	109.5
C2—C7—C8	109.19 (19)	H15A—C15—H15B	109.5
C6—C7—C8	129.2 (2)	C13—C15—H15C	109.5
N1—C8—C7	115.26 (14)	H15A—C15—H15C	109.5
N1—C8—O2	111.73 (16)	H15B—C15—H15C	109.5
C7—C8—O2	102.67 (15)	C8—N1—C9	122.95 (13)
N1—C8—H8	109.0	C8—N1—H1	118.5
C7—C8—H8	109.0	C9—N1—H1	118.5
O2—C8—H8	109.0	C1—O2—C8	110.39 (16)
C14—C9—C10	118.71 (16)	C10—O3—H3A	109.5
			
O1—C1—C2—C7	−179.2 (2)	N1—C9—C10—O3	1.9 (3)
O2—C1—C2—C7	−0.1 (2)	C14—C9—C10—C11	0.2 (3)
O1—C1—C2—C3	−0.4 (4)	N1—C9—C10—C11	−178.11 (17)
O2—C1—C2—C3	178.76 (19)	O3—C10—C11—C12	−178.1 (2)
C7—C2—C3—C4	0.8 (3)	C9—C10—C11—C12	2.0 (3)
C1—C2—C3—C4	−177.9 (2)	C10—C11—C12—C13	−1.9 (3)
C2—C3—C4—C5	0.5 (3)	C11—C12—C13—C14	−0.3 (3)
C3—C4—C5—C6	−1.5 (4)	C11—C12—C13—C15	180.0 (2)
C4—C5—C6—C7	1.0 (3)	C10—C9—C14—C13	−2.5 (3)
C3—C2—C7—C6	−1.3 (3)	N1—C9—C14—C13	175.70 (19)
C1—C2—C7—C6	177.66 (17)	C12—C13—C14—C9	2.6 (3)
C3—C2—C7—C8	179.96 (17)	C15—C13—C14—C9	−177.7 (2)
C1—C2—C7—C8	−1.11 (19)	C7—C8—N1—C9	−162.60 (18)
C5—C6—C7—C2	0.3 (3)	O2—C8—N1—C9	80.7 (2)
C5—C6—C7—C8	178.81 (19)	C14—C9—N1—C8	−10.7 (3)
C2—C7—C8—N1	−120.00 (19)	C10—C9—N1—C8	167.55 (18)
C6—C7—C8—N1	61.4 (3)	O1—C1—O2—C8	−179.57 (18)
C2—C7—C8—O2	1.72 (17)	C2—C1—O2—C8	1.21 (19)
C6—C7—C8—O2	−176.92 (18)	N1—C8—O2—C1	122.30 (16)
C14—C9—C10—O3	−179.76 (17)	C7—C8—O2—C1	−1.79 (17)

### Hydrogen-bond geometry (Å, °)

**Table:**

*D*—H···*A*	*D*—H	H···*A*	*D*···*A*	*D*—H···*A*
O3—H3A···O1^i^	0.82	1.95	2.767 (2)	173
N1—H1···O2^ii^	0.86	2.78	3.5593 (19)	152

Symmetry codes: (i) −*x*+1/2, *y*+1/2, *z*+1/2; (ii) *x*−1/2, −*y*+1/2, *z*.
